# 替吉奥治疗晚期非小细胞肺癌三线及以上患者的疗效分析

**DOI:** 10.3779/j.issn.1009-3419.2018.06.03

**Published:** 2018-06-20

**Authors:** 一 尹, 标 吴, 章洲 黄, 武 庄, 振武 徐, 诚 黄, 韵坚 黄, 晶 张

**Affiliations:** 350014 福州，福建省肿瘤医院，福建医科大学附属肿瘤医院肿瘤内科 Department of Medical Oncology, Fujian Cancer Hospital Fujian Medical University Cancer Hospital, Fuzhou 350014, China

**Keywords:** 替吉奥, 肺肿瘤, 化疗, 药物耐药, S-1, Lung neoplasms, Chemotherapy, Drug resistance

## Abstract

**背景与目的:**

晚期非小细胞肺癌一二线治疗后进展，目前尚无标准的治疗方案，替吉奥作为安全低毒的第三代氟脲嘧啶衍生物，对肺癌具有一定疗效，本研究旨在探讨替吉奥在晚期三线及以上非小细胞肺癌患者中的疗效。

**方法:**

回顾性分析105例2014年1月-2017年4月我院收治的使用替吉奥单药或联合方案治疗三线及以上晚期非小细胞肺癌患者的临床资料，替吉奥使用方法：体表面积 < 1.25 m^2^，每日80 mg；1.25 m^2^-1.5 m^2^，每日100 mg；≥1.5 m^2^，每日120 mg，分2次口服，连续使用1 d-14 d，21 d为1个周期。可联合铂类或其他第三代化疗药物。每周期根据实体瘤疗效评价标准（Response Evaluation Criteria in Solid Tumors, RECIST）1.1标准评价近期疗效，采用*Kaplan-Meier*方法统计生存数据。

**结果:**

替吉奥单药治疗42例，联合用药63例，中位治疗线数4（3-11），中位周期数2（1-14）。无完全缓解患者，部分缓解患者4例，疾病稳定患者34例，疾病进展患者67例。客观有效率3.81%，疾病控制率36.19%。中位无进展生存期1.90个月（95%CI: 0.67-10.83），单药或联合治疗疗效相似（疾病控制率：28.56% *vs* 41.27%，*P*=0.185），肝转移患者预后更差（中位无进展生存期：1.40个月*vs* 1.93个月，*P*=0.042）。

**结论:**

在晚期非小细胞肺癌患者三线及以上抗肿瘤治疗中，替吉奥具有一定疗效，联合用药不能进一步提高疗效，替吉奥单药可作为多程治疗后患者的选择之一。

## Abstract (en)

**Background and objective:**

There is no standard treatment for advanced non-small cell lung cancer (NSCLC) after the failure of two lines of chemotherapy, S-1 as the third generation of fluorouracil derivate with well safety and low toxicity, presented some efficacy in lung cancer treatment. The aim of this study is to explore the efficacy of S-1 for advanced NSCLC patients treated with two or more prior chemotherapy regimens.

**Methods:**

We performed a retrospective analysis of 105 NSCLC patients treated with S-1 monotherapy or S-1 contained chemotherapy as the third or more line of treatment in our hospital from January 2014 to April 2017. S-1 was administrated orally twice daily for 2 weeks, followed by one week of rest, the dose of drug was determined by body surface area (< 1.25 m^2^, 80 mg/d; 1.25 m^2^-1.5 m^2^, 100 mg/d; ≥1.5 m^2^, 120 mg/d), platinum or the third-generation chemotherapy drugs could be combinedly used. Clinical response was assigned every cycle according to Response Evaluation Criteria in Solid Tumors (RECIST) version 1.1, *Kaplan-Meier* analysis was used to estimate progression-free survival (PFS).

**Results:**

42 patients received S-1 monotherapy, the other 63 patients received combined regimens, the median treatment line was 4 (3-11) and the median treatment cycle was 2 (1-14). No complete response (CR) were observed, there were 4 patients with partial response (PR), 34 patients with stable disease (SD) and 67 patients with progressive disease (PD), the objective response rate (ORR) was 3.81%, disease control rate (DCR) was 36.19%. The median PFS was 1.90 months (0.67 months-10.83 months), no difference between monotherapy and combined group (DCR: 28.56% *vs* 41.27%, *P*=0.185), the liver metastasis showed poorer PFS (1.40 months *vs* 1.93 months, *P*=0.042).

**Conclusion:**

S-1 presented some activity in advanced NSCLC treated with more than two lines of treatment. The addition of other drugs cannot improve efficacy. S-1 monotherapy can be used as a choice for heavily-treated patients.

肺癌是目前发病率及死亡率最高的恶性肿瘤，85%左右为非小细胞肺癌（non-small cell lung cancer, NSCLC）^[1]^，大多数患者在诊断初或治疗过程中进展为晚期患者，经过标准的一二线治疗后，仍有部分患者身体状况较好，治疗积极性高，需要接受三线乃至更多线的治疗，并且随着低毒化疗药物的不断出现，使得患者的治疗有了更多的可能性，但目前尚无标准的三线治疗方案。替吉奥胶囊为第三代氟脲嘧啶衍生物口服抗癌药物，由替加氟、吉美嘧啶和奥替拉西三药联合组成^[2]^，替加氟在人体代谢后产生5-氟尿嘧啶（5-Fluorouracil, 5-Fu）发挥抗癌作用，吉美嘧啶的加入可以使得5-Fu在肺癌组织中的有效浓度及持续时间得到极大提升，媲美5-Fu持续泵入的效果，同时奥替西拉能够有效地抑制肠道粘膜内乳清酸核糖转移酶，减轻肠道不良反应^[3]^。替吉奥具有安全、方便、经济、不需住院及输液等优势，尤其适合多线治疗后、化疗耐受性下降的患者使用，既往研究证实其对NSCLC具有一定疗效并且不良反应轻，可以媲美一线治疗药物^[4-6]^，但对于多程治疗后患者的研究样本量普遍较小，且研究结论存在争议^[7-9]^。本研究评价替吉奥在多线治疗后晚期NSCLC患者中的疗效。

## 1 资料与方法

### 1.1 患者与资料

回顾性分析2014年1月-2017年4月我院收治的使用替吉奥单药或联合方案治疗三线及以上晚期NSCLC患者的临床资料。纳入标准：①诊断经组织病理证实；②根据美国癌症联合委员会（American Joint Committeeon Cancer, AJCC）第七版分期为Ⅳ期；③美国东部肿瘤协作组（Eastern Cooperative Oncology Group, ECOG）体力状况评分为0-2分；④无其他恶性肿瘤病史；⑤心、肝、肾等脏器功能基本正常；⑥接受过标准一线二线方案化疗，表皮生长因子受体（epidermal growth factor receptor, *EGFR*）突变型的患者接受过第一代表皮生长因子受体酪氨酸激酶抑制剂（EGFR tyrosine kinase inhibitor, EGFR-TKI）治疗（部分患者接受过第一、三代TKI药物），间变性淋巴瘤激酶（anaplastic lymphoma kinase, *ALK*）突变患者接受过克唑替尼治疗（未接受过第二、三代ALK抑制剂）；⑦有可测量的靶病灶；⑧至少完成化疗1周期。排除标准：①联合EGFR-TKI、ALK抑制剂、多靶点抑制剂、抗血管生成等靶向药物者；②失访或自行中断治疗者。

共入选105例患者。男性75例（71.4%），女性30例（28.6%）；中位年龄54岁（33岁-75岁），65岁及以上患者15例（14.29%）；ECOG 0分-1分80例（76.19%），2分25例；鳞癌36例，腺癌56例，腺鳞癌3例，大细胞癌1例，未分类的低分化癌9例。56例检测EGFR状态，13例敏感突变（23.21%），43例无突变。41例患者检测ALK状态，3例患者阳性（7.32%）。替吉奥单药治疗42例，联合用药63例，包括铂类药物33例（顺铂16例、卡铂17例）、第三代化疗药物30例（多西他赛17例、紫杉醇6例、吉西他滨4例、伊立替康3例），ECOG 2分的患者均接受替吉奥单药治疗。中位治疗线数4（3-11），60例（57.14%）患者为四线及以上的治疗，中位周期数2（1-14）。中位内脏及骨转移部位个数2（1-6），其中肝转移17例（16.2%），脑转移32例（30.5%）（表 1）。

**1:** 105例晚期NSCLC患者的基本特征 Clinical characteristics of 105 patients with advanced NSCLC

Clinical characteristics	*n* (%)
Gender	
Male	30 (28.57)
Female	75 (71.43)
Age (yr)	
< 65	90 (85.71)
≥65	15 (14.29)
ECOG	
0-1	80 (76.19)
2	25 (23.81)
Pathological subtype	
Squamous	36 (34.28)
Non-squamous	69 (65.71)
EGFR	
Positive	13 (12.38)
Negative	43 (40.95)
Unknow	49 (46.67)
ALK	
Positive	3 (2.86)
Negative	38 (36.19)
Unknow	64 (60.95)
S-1 monotherapy	42 (40.00)
Combined-chemotherapy	63 (60.00)
Platinum	33 (31.43)
Docetaxel	17 (16.19)
Paclitaxel	6 (5.71)
Gemcitabine	4 (3.81)
Irinotecan	3 (2.86)
Therapy lines	
3	45 (42.86)
≥4	60 (57.14)
Metastasis number	
1-2	66 (62.86)
3-6	39 (37.14)
Liver metastasis	17 (16.19)
Brain metastasis	32 (30.48)
ECOG: Eastern Cooperative Oncology Group; NSCLC: non-small cell lung cancer; EGFR: epidermal growth factor receptor.	

### 1.2 治疗方案

替吉奥使用方法：体表面积 < 1.25 m^2^，每日80 mg；1.25 m^2^-1.5 m^2^，每日100 mg；≥1.5 m^2^，每日120 mg，分2次口服，连续口服1 d-14 d，21 d为1个周期。联合用药使用方法：顺铂25 mg/m^2^第1-3天静脉滴注，卡铂按曲线下面积（area under the curve, AUC）=5计算剂量，第1天静脉滴注，多西他赛60 mg/m^2^、紫杉醇175 mg/m^2^，第1天静脉滴注，吉西他滨1.0 g/m^2^、伊立替康100 mg/m^2^第1、8天静脉滴注，均为21天为1个周期。治疗同时可给予5-羟色胺受体抑制剂、糖皮质激素预防性止吐，并重组人粒细胞集落刺激因子纠正骨髓抑制等辅助治疗。治疗至疾病进展或毒副反应不能耐受终止。如患者存在脑转移，给予脑部转移瘤放射治疗。

### 1.3 治疗评价

每周期治疗后对病灶部位进行胸部计算机断层扫描（computed tomography, CT）、腹部磁共振成像（magnetic resonance imaging, MRI）/CT、头颅MRI等影像学检查，根据实体瘤疗效评价标准（Response Evaluation Criteria in Solid Tumors, RECIST）1.1评价近期疗效，包括完全缓解（complete response, CR）、部分缓解（partial response, PR）、疾病稳定（stable disease, SD）、疾病进展（progressive disease, PD）、客观缓解率（objective response rate, ORR）：CR+PR患者所占的比例、以及疾病控制率（disease control rate, DCR）：CR+PR+SD患者所占的比例。无进展生存时间（progression-free survival, PFS）定义为自用药第1天起至疾病进展或随访截止日。根据国立癌症研究所毒性判定标准（Common Terminology Criteria for Adverse Events, CTC）4.0每周期对患者进行不良反应评价。

### 1.4 统计学分析

采用SPSS 23.0统计软件，临床特征与近期疗效的关系采用卡方检验，*Kaplan-Meier*方法统计生存数据，*Log-rank*法进行单因素分析。*P* < 0.05认为有统计学差异。采用graphpad prism 5.0软件绘制生存曲线图。

## 2 结果

### 2.1 近期疗效

无CR患者，PR患者4例、SD患者34例、PD患者67例，ORR 3.81%，DCR 36.19%。DCR与患者性别、年龄（< 65岁或≥65岁）、病理类型（鳞癌/非鳞癌）、*EGFR*突变状态、治疗线数（3线或以上）、转移部位数（2个或以上）、肝转移、脑转移之间均未发现统计学差异，联合用药的疗效亦无明显改善（28.56% *vs* 41.27%, *P*=0.185）（表 2）。替吉奥单药治疗的患者，PR 1例、SD 11例、PD 30例，ORR 2.38%，DCR 28.57%，联合治疗的患者，PR 3例、SD 23例，PD 37例，ORR 4.76%，DCR 41.27%，联合药物的类型（铂类或非铂类）不影响疗效（39.39% *vs* 43.33%, *P*=0.751）。疗效与上述各临床特征之间均未发现统计学差异（表 3，表 4）。

**2:** 替吉奥（单药和联合）治疗三线及以上晚期NSCLC患者的疗效与临床特征分析 The S-1 (mono- and combined therapy) efficacy analysis of clinical characteristics of advanced NSCLC patients treated with more than two lines of chemotherapy

Characteristic	*n*	DCR	*X*^2^	*P*	PFS (months)	*P*
		*n* (%)			Median (95%CI)	
Gender			1.996	0.158		0.932
Male	75	24 (32.00)			1.87 (1.43-2.37)	
Female	30	14 (46.67)			1.90 (1.196-2.538)	
Age (yr)			0.122	0.727		0.549
< 65	90	36 (40.00)			1.90 (1.49-2.31)	
≥65	15	2 (13.33)			1.63 (0.00-4.15)	
Pathological subtype			0.753	0.385		0.367
Squamous	36	11 (30.56)			1.73 (0.61-2.86)	
Non-squamous	69	27 (39.13)			1.93 (1.24-2.63)	
EGFR				0.747		0.486
Positive	13	4 (30.77)			1.83 (1.03-2.64)	
Negative	43	17 (39.53)			1.87 (1.01-2.72)	
Combined-chemotherapy			1.760	0.185		0.842
Yes	63	36 (41.27)			2.03 (1.55-2.52)	
No	42	12 (28.57)			1.63 (0.89-2.37)	
Therapy lines			1.241	0.265		0.081
3	45	19 (42.22)			2.03 (1.66-2.08)	
≥4	60	19 (31.67)			1.87 (0.68-3.39)	
Metastasis number			0.002	0.962		0.252
1-2	66	24 (36.36)			1.90 (1.24-2.56)	
3-6	39	14 (35.90)			1.83 (1.06-2.61)	
Liver metastasis			3.020	0.082		0.042
Yes	17	3 (17.65)			1.40 (0.73-2.07)	
No	88	24(39.78)			1.93 (1.32-2.55)	
Brain metastasis			0.066	0.798		0.138
Yes	32	11 (36.99)			1.90 (1.24-2.49)	
No	73	27 (34.38)			1.87 (1.35-2.45)	
EGFR: epidermal growth factor receptor; DCR: disease control rate.						

**3:** 替吉奥单药治疗三线及以上晚期NSCLC患者的疗效与临床特征分析 The S-1 (monotherapy) efficacy analysis of clinical characteristics of advanced NSCLC patients treated with more than two lines of chemotherapy

Characteristic	*n*	DCR	*X*^2^	*P*	PFS (month)	*P*
		*n* (%)			Median (95%CI)	
Gender			0.210	0.359		0.115
Male	35	11 (31.43)			1.03 (0.26-1.80)	
Female	7	1 (14.29)			1.73 (0.73-2.74)	
Age (yr)			0.588	0.443		0.383
< 65	36	10 (27.78)			1.53 (0.46-2.61)	
≥65	6	2 (33.33)			1.63 (0.00-7.07)	
Pathological subtype			0.131	0.717		0.240
Squamous	14	3 (21.43)			1.03 (0.61-1.46)	
Non-squamous	28	9 (32.14)			1.77 (0.64-2.89)	
EGFR				1.000		0.452
Positive	16	2(12.50)			1.63 (1.18-2.09)	
Negative	7	5(71.43)			1.33 (0.56-2.10)	
Therapy lines			0.131	0.717		0.285
3	14	6 (42.86)			1.53 (0.43-2.63)	
≥4	28	6 (21.43)			1.63 (0.89-2.37)	
Metastasis number			0.131	0.717		0.234
1-2	28	9 (32.14)			1.73 (0.87-2.60)	
3-6	14	3 (21.43)			1.63 (0.89-2.37)	
Liver metastasis			2.216	0.145		0.001
Yes	12	1 (8.33)			1.00 (0.66-1.33)	
No	30	11 (36.67)			2.40 (1.24-3.56)	
Brain metastasis			1.185	0.276		0.122
Yes	10	1 (10.00)			1.03 (0.00-2.22)	
No	32	11 (34.38)			1.63 (0.99-2.28)	

**4:** 替吉奥联合治疗三线及以上晚期NSCLC患者的疗效与临床特征分析 The S-1 (combined regimen) efficacy analysis of clinical characteristics of advanced NSCLC patients treated with more than two lines of chemotherapy

Characteristic	*n*	DCR	*X*^2^	*P*	PFS (month)	*P*
		*n* (%)			Median (95%CI）	
Gender			3.477	0.062		0.206
Male	40	13 (32.50)			1.90 (1.59-2.21)	
Female	23	13 (56.25)			2.30 (1.20-3.40)	
Age (yr)			0.025	0.875		0.588
< 65	54	26 (48.15)			1.93 (1.45-2.41)	
≥65	9	0			2.30 (0.45-4.15)	
Pathological subtype			0.336	0.562		0.805
Squamous	22	8 (36.36)			1.90 (1.17-2.63)	
Non-squamous	41	18 (43.90)			2.03 (1.16-2.91）	
EGFR						0.649
Positive	6	2 (33.33)		> 0.999	1.83 (1.04-2.63)	
Negative	27	12 (44.44)			1.90 (0.83-2.97)	
Therapy lines			0.011	0.916		0.198
3	31	13 (41.94)			2.30 (0.88-3.72)	
≥4	32	13 (40.63)			1.90 (1.53-2.27)	
Metastasis number			0.127	0.721		0.629
1-2	38	15 (39.47)			1.90 (0.89-2.91)	
3-6	25	11 (44.00)			2.03 (1.54-2.52）	
Liver metastasis			< 0.001	> 0.999		0.463
Yes	5	2 (40.22)			2.60 (0.60-3.60)	
No	58	23 (39.66)			1.90 (1.37-2.43)	
Brain metastasis			0.336	0.562		0.494
Yes	22	10 (45.45)			2.03 (1.23-2.84)	
No	41	16(39.02)			1.93 (1.26-2.61)	
Combined agent			0.101	0.751		0.078
Platinum	33	13 (39.39)			1.83 (1.01-2.66)	
Others	30	13 (43.33)			2.60 (1.57-3.63)	

### 2.2 生存分析

截止2017年10月，中位随访19.33个月（6.23-46.40），100例患者出现进展，中位PFS 1.90个月（0.67-10.83）（图 1）。单因素分析显示患者性别、年龄、病理类型（图 2）、EGFR状态、治疗线数、联合化疗与否（图 3），治疗前内脏或骨转移部位个数、脑转移等均对PFS无影响，而肝转移患者预后更差（1.40个月*vs* 1.93个月，*P*=0.042）（表 2，图 4）。替吉奥单药治疗的患者，PFS为1.63个月（95%CI: 0.89-2.37），肝转移是影响PFS的单因素（1.00个月*vs* 2.40个月，*P*=0.001），其他临床特征与PFS之间无统计学关联（表 3）。联合治疗的患者，PFS为2.03个月（95%CI: 1.55-2.52），单因素分析未发现有统计学差异的临床特征，联合铂类或者非铂类药物亦不影响PFS（1.83个月*vs* 2.60个月，*P*=0.078）（图 5，表 4）。

**1:**
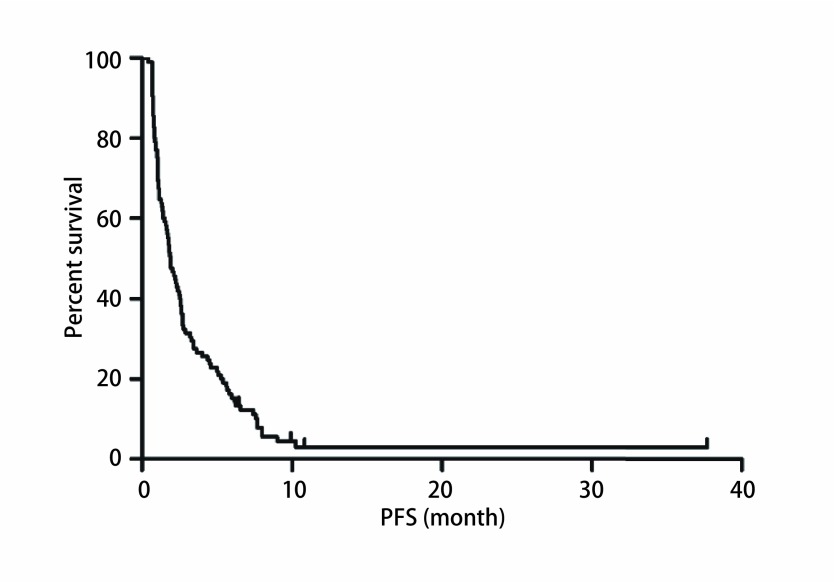
105例晚期NSCLC患者接受替吉奥化疗的PFS PFS of 105 advanced NSCLC patients treated with S-1.

**2:**
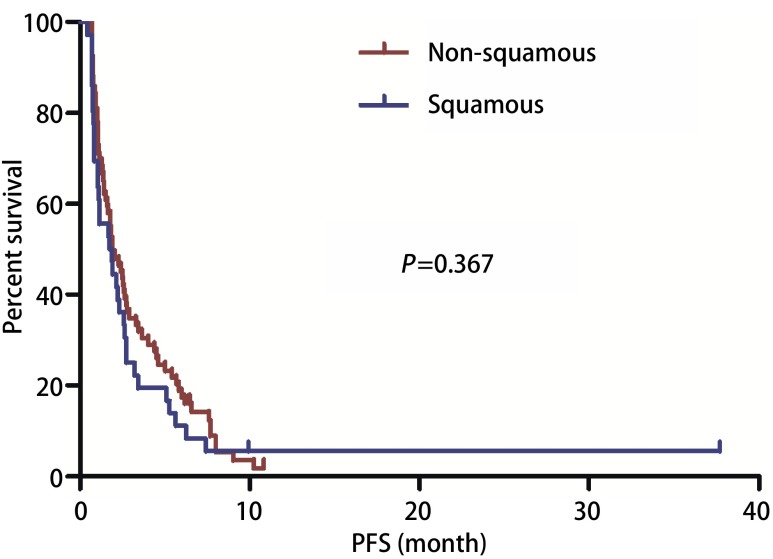
替吉奥治疗不同病理类型晚期NSCLC患者的PFS PFS of advanced NSCLC patients according to pathological subtype. PFS: progression-free survival.

**3:**
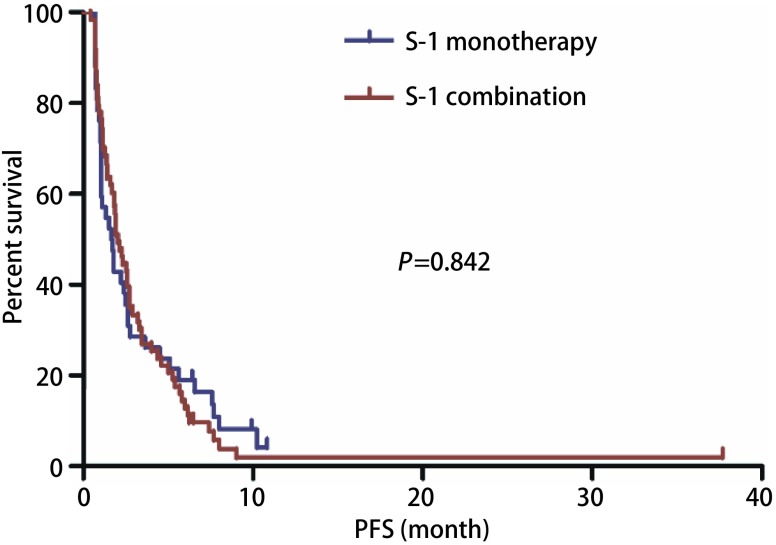
替吉奥单药和联合方案治疗晚期NSCLC患者的PFS PFS of advanced NSCLC patients according to S-1 monotherapy /combination

**4:**
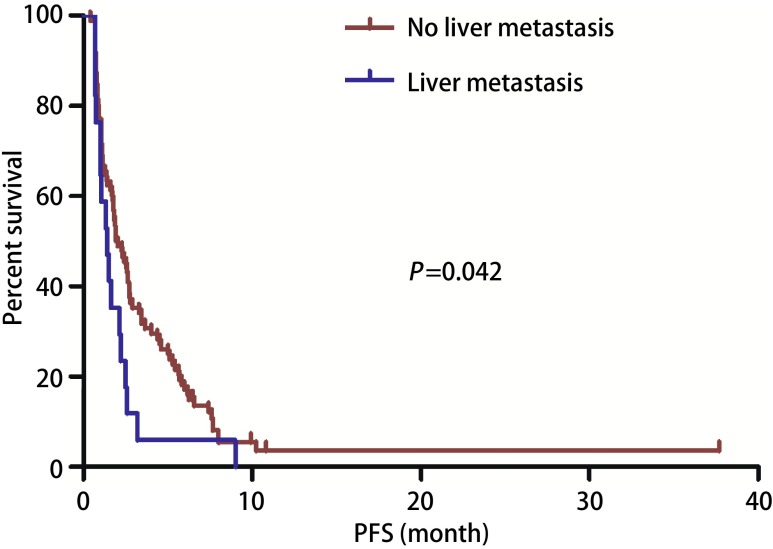
肝转移和非肝转移NSCLC患者的PFS PFS of advanced NSCLC patients according to liver metastasis

**5:**
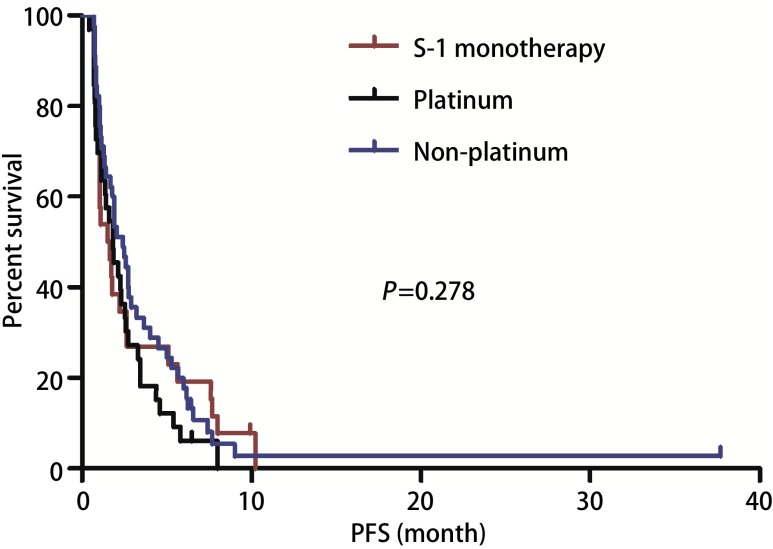
替吉奥单药、联合铂类和联合非铂类治疗晚期NSCLC患者的PFS PFS of advanced NSCLC patients according to S-1 monotherapy/platinum-combination/non-platinum combination

### 2.3 不良反应

患者主要的不良反应为恶心呕吐、白细胞减少及皮肤色素沉着。替吉奥单药治疗的恶心呕吐22例（52.38%），均为1级-2级；皮肤色素沉着5例（11.90%），均为1级-2级；白细胞减少23例（54.76%），其中3级-4级为5例（11.90%）。未观察到因不良反应不能耐受而中止治疗或减量者。联合用药组47例发生恶心呕吐，其中5例（7.93%）患者为3级，其余均为1级-2级，45例（71.42%）患者发生白细胞减少，其中3级-4级发生率为36.51%（23/63）。6例（9.52）患者因白细胞减少减量。皮肤色素沉着4例（6.35%），均为1级-2级。

## 3 讨论

本研究发现替吉奥在三线及以上晚期NSCLC患者中具有一定疗效，超过30%的患者得到疾病控制，中位PFS约2个月。既往研究^[7-10]^显示在二线及以上治疗的患者中，替吉奥单药或联合治疗的DCR为14.3%-50.0%，中位PFS为2个月-3个月，同我们的研究类似。

目前，多线治疗中联合化疗能否进一步提高疗效存在争议，Kim等^[9]^报道的一项Ⅱ期研究显示替吉奥联合伊立替康或多西他赛治疗三线及以上患者的ORR为0，DCR为14.3%，疗效不佳。马迪等^[11]^进行的一项回顾性分析显示替吉奥联合奈达铂二线或多线治疗晚期NSCLC的DCR为64.7%，中位PFS为3.0个月，疗效较好。但这两项研究病例数均较少（< 20例），且第二项研究部分患者为二线治疗，不能完全适用三线及以上患者。我们的研究显示，联合用药的DCR高于单药治疗（41.27% *vs* 28.56%），但未达统计学差异（*P*=0.185），一方面可能与样本量不足有关；另一方面，患者经过多线治疗化疗敏感性下降，联合用药带来获益可能较为有限，结合患者PFS获益更加微弱（2.03个月*vs* 1.63个月，*P*=0.842）、联合治疗的不良反应更大，我们认为，联合治疗在多线治疗中的优势不足，单药治疗是更值得考虑的方案。

既往研究发现替吉奥对不同病理类型的患者疗效存在差异，Hisamatsu等^[10]^报道的一项回顾性研究纳入了71例接受替吉奥单药化疗的二、三线患者，结果显示鳞癌患者ORR为0，而腺癌患者ORR为11%（*P*=0.33）。Tomita^[12]^的一项回顾性研究也显示，在54例多线治疗的患者中，腺癌的DCR高于鳞癌患者（57.9% *vs* 20.0%, *P*=0.013），可能的解释包括：鳞癌组织中胸苷酸合成酶（thymidylate synthases, TS）含量较高，而较高水平的TS对5-FU及其衍生物会产生耐药作用。但Yamamoto等^[13]^对两项Ⅱ期临床研究进行荟萃分析得出不同的结论，该研究共110例患者使用S-1联合顺铂作为一线治疗，结果显示病理类型不影响患者疗效（ORR：鳞47.6% *vs*非鳞38.2%）。我们的研究同样发现无论是单药或联合治疗，替吉奥对不同病理类型的患者疗效类似，考虑可能三线治疗后药物疗效均明显差于一线治疗，进而病理类型对药物疗效影响不明显。

包括IPSS^[14]^在内的许多研究认为在一线化疗中，*EGFR*敏感突变的患者的疗效好于EGFR野生型者，秦娜等^[15]^报道的一项回顾性分析显示，*EGFR*突变组和野生型组的DCR为（84.0% *vs* 60.4%, *P*=0.001），并且*EGFR*突变是影响PFS的独立因素（HR=0.654, 95%CI: 0.470-0.909, *P*=0.012），而本研究未观察到这种现象，Inagaki等^[16]^进行了一项纳入283例患者的多中心回顾性研究，同样发现多线治疗后，EGFR状态不影响替吉奥疗效，我们认为肿瘤患者的多线化疗同一线化疗存在区别，同样由于化疗敏感性下降，多线治疗对患者的选择性较弱，EGFR状态对患者疗效影响不明显。

生存分析发现，肝转移患者预后较差，这一发现在既往的研究^[17]^中也得到证实，可能与肝脏血供丰富转移瘤易于生长扩散及影响药物代谢等因素有关。治疗线数及转移部位个数不影响替吉奥疗效，提示多程治疗后的患者均可使用该药物。我们的脑转移患者均接受过脑部放疗，而脑转移与否不影响患者的DCR与PFS，提示在脑转移瘤放疗的情况下，脑转移患者亦可使用。

综上所述，替吉奥在经过多线抗肿瘤治疗后的晚期NSCLC治疗中显示出一定疗效，疗效不受病理类型、EGFR状态、治疗线数、脑转移等因素影响，联合用药不能进一步提高疗效。单药口服化疗药物毒性更低，更为经济方便，有利于提高患者生活质量及治疗依从性，减轻治疗负担，是目前多线治疗后的较好选择。由于该研究为回顾性，存在诸多局限性，需要更多的研究进一步证实。
